# Supplementary figures and images for: Sustained Ca2+ mobilizations: A quantitative approach to predict their importance in cell-cell communication and wound healing
Source: PLoS One. 2019 Apr 24;14(4):e0213422. doi: 10.1371/journal.pone.0213422 (PMC6481807; doi:10.1371/journal.pone.0213422)

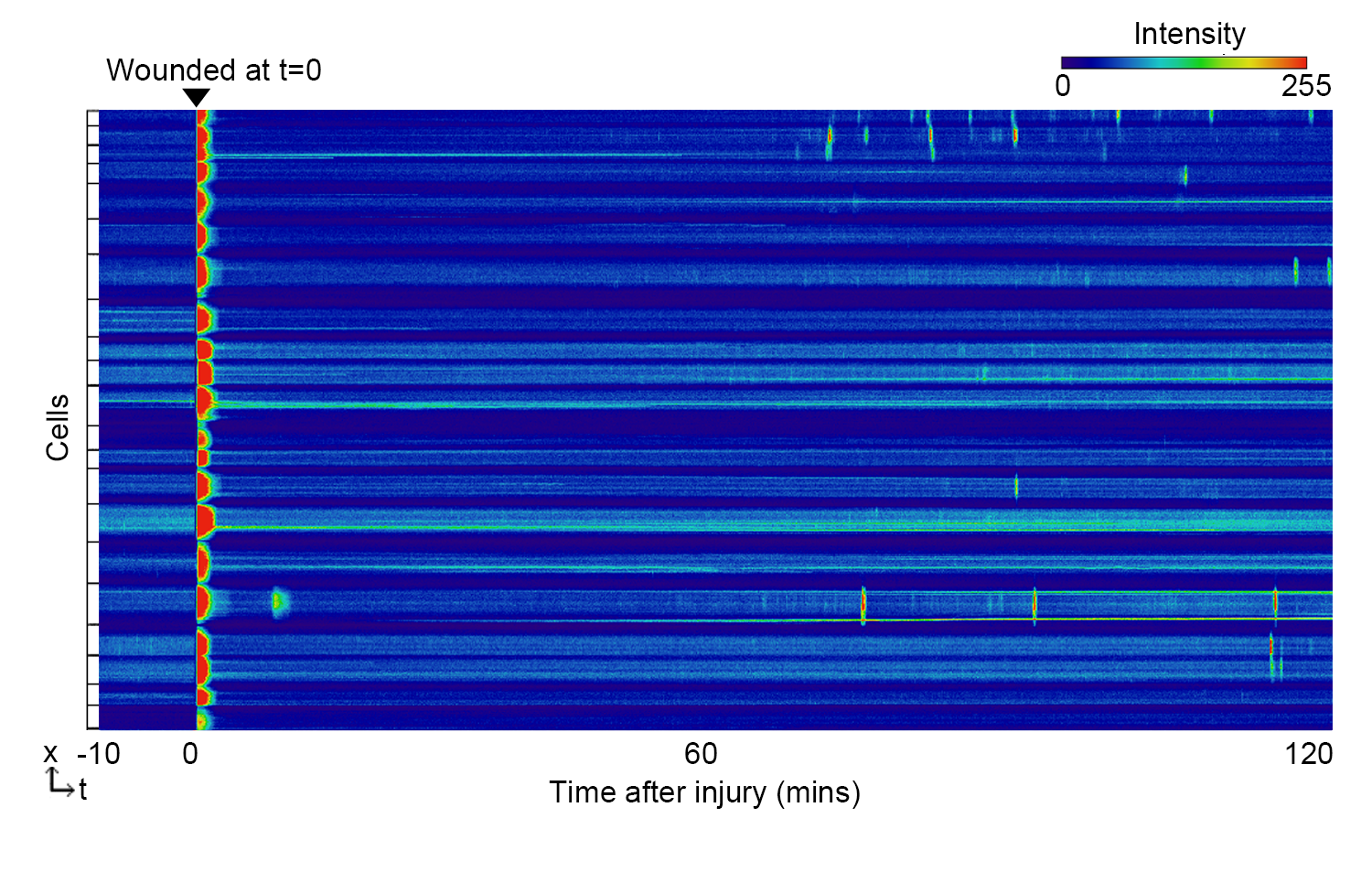

Supplement: S1 Fig — Compared to the kymographs made from cells at the wound edge (LE), the Ca2+ response showed less intensity. Brackets on the left and each horizontal line represent activity of a single cell (n = 7). (TIF) [file pone.0213422.s001.tif]

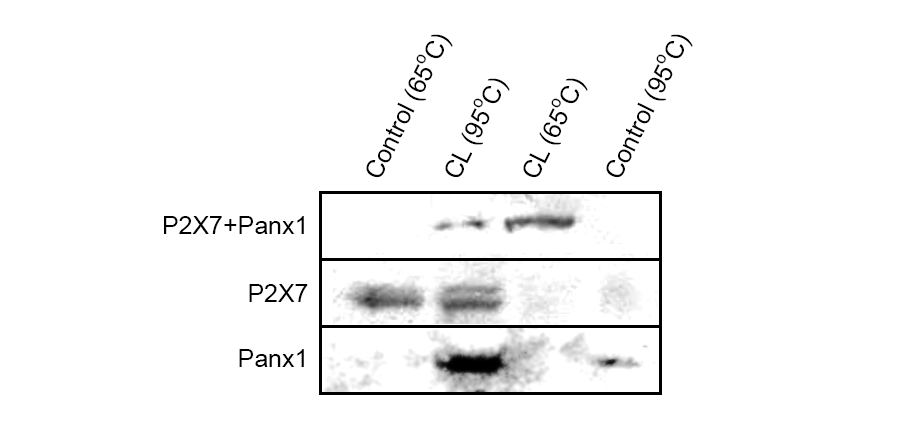

Supplement: S2 Fig — HCLE cells were cultured until confluent, and cross-linking was performed with formaldehyde in situ, as previously described [12]. Each crosslinked experimental sample (labeled “CL”) and its corresponding control were heated at two different temperature settings: 65°C (to maintain crosslinks) and 95°C (to disrupt crosslinks). Both CL lanes displayed the crosslinked P2X7+ pannexin1 protein product, with the CL (95°C) lane verifying the composition crosslinked protein product. (n = 3). (TIF) [file pone.0213422.s002.tif]
